# 4,6-Di­bromo-*N*-{3-[(4,6-di­bromo-2,3-di­methyl­phenyl)imino]butan-2-yl­idene}-2,3-di­methyl­aniline

**DOI:** 10.1107/S1600536813023921

**Published:** 2013-09-04

**Authors:** Lina Huang, Zhengyin Du, Wei Liu, Fushou Che

**Affiliations:** a Key Laboratory of Eco-Environment-Related Polymer Materials of the Ministry of Education, Key Laboratory of Polymer Materials of Gansu Province, College of Chemistry & Chemical Engineering, Northwest Normal University, Lanzhou 730070, People’s Republic of China

## Abstract

The title compound, C_20_H_20_Br_4_N_2_, is a product of the condensation reaction of 4,6-di­bromo-2,3-di­methyl­aniline and butane-2,3-dione. The mol­ecule has a center of symmetry at the mid-point of the central C—C bond. The dihedral angle between the benzene ring and the 1,4-di­aza­butadiene plane is 78.3 (2)°. Niether hydrogen bonding nor aromatic stacking is observed in the crystal structure.

## Related literature

For applications of di­imine–metal catalysts, see: Johnson *et al.* (1995 ▶). For related structures, see: Gao *et al.* (2012 ▶); Sun *et al.* (2012 ▶); Popeney *et al.* (2012 ▶); Shi *et al.* (2012 ▶); Zhang & Ye (2012 ▶); Killian *et al.* (1996 ▶); Yuan *et al.* (2005 ▶, 2011 ▶).
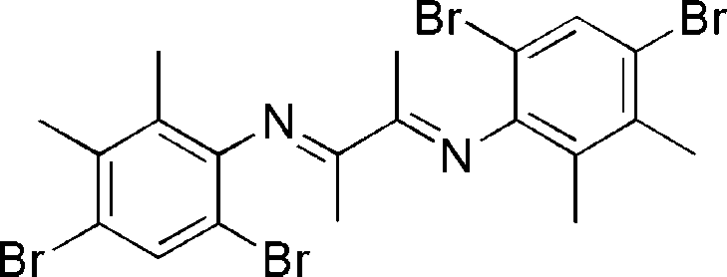


## Experimental

### 

#### Crystal data

C_20_H_20_Br_4_N_2_
*M* *_r_* = 607.98
Monoclinic, 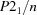

*a* = 5.5582 (6) Å
*b* = 12.8881 (15) Å
*c* = 14.8377 (11) Å
β = 98.782 (8)°
*V* = 1050.43 (19) Å^3^
*Z* = 2
Cu *K*α radiation
μ = 9.40 mm^−1^
*T* = 150 K
0.29 × 0.17 × 0.16 mm

#### Data collection

Agilent SuperNova (Dual, Cu at zero, Eos) diffractometer
Absorption correction: multi-scan (*CrysAlis PRO*; Agilent, 2013 ▶) *T*_min_ = 0.688, *T*_max_ = 1.000
4675 measured reflections
1921 independent reflections
1777 reflections with *I* > 2σ(*I*)
*R*_int_ = 0.029

#### Refinement

*R*[*F* ^2^ > 2σ(*F* ^2^)] = 0.044
*wR*(*F* ^2^) = 0.135
*S* = 1.11
1921 reflections
121 parameters
H-atom parameters constrained
Δρ_max_ = 0.70 e Å^−3^
Δρ_min_ = −1.04 e Å^−3^

### 

Data collection: *CrysAlis PRO* (Agilent, 2013 ▶); cell refinement: *CrysAlis PRO*; data reduction: *CrysAlis RED* (Agilent, 2013 ▶); program(s) used to solve structure: *SUPERFLIP* (Palatinus & Chapuis, 2007 ▶); program(s) used to refine structure: *OLEX2* (Dolomanov *et al.*, 2009 ▶); molecular graphics: *OLEX2*; software used to prepare material for publication: *OLEX2*.

## Supplementary Material

Crystal structure: contains datablock(s) I. DOI: 10.1107/S1600536813023921/rk2409sup1.cif

Structure factors: contains datablock(s) I. DOI: 10.1107/S1600536813023921/rk2409Isup2.hkl

Supplementary material file. DOI: 10.1107/S1600536813023921/rk2409Isup3.cml

Additional supplementary materials:  crystallographic information; 3D view; checkCIF report

## Figures and Tables

**Fig. 1.**
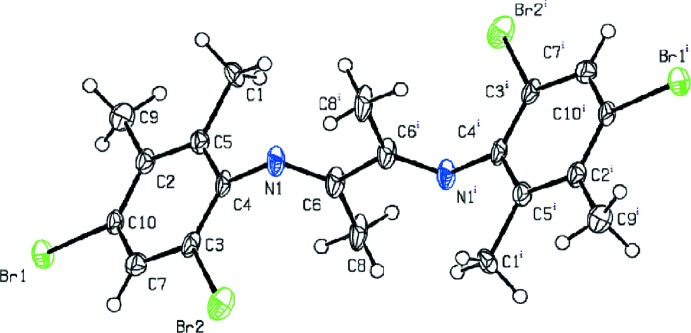
Molecular structure of title compound with the atom numbering scheme. Displacement ellipsoids are shown at the 50% probability level. H atoms are presented as a small spheres of arbitrary radius. Symmetry code: (i) -x+1, -y+1, -z+1.

**Fig. 2.**
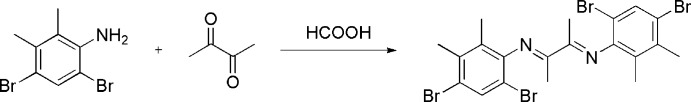
A condensation reaction of 2,3–butanedione and 4,6–dibromo-2,3–dimethylaniline.

## supplementary crystallographic information

### 1. Comment

Since Brookhart and co–workers discovered nickel and palladium(II) aryl–substituted α–diimine catalysts for olefin polymerization (Johnson *et al.*, 1995), late transition metal catalysis has attracted increasing attention due to their high functional group tolerance and their ability to produce branched or dendritic polymer (Gao *et al.*, 2012; Sun *et al.*, 2012; Popeney *et al.*, 2012; Shi *et al.*, 2012; Zhang & Ye, 2012; Killian *et al.*, 1996; Yuan *et al.*, 2005;2011). In this study, we designed and synthesized the title compound as abidentate ligand (Fig. 1).

The title molecule placed in center of symmetry (middle of C6—C6^i^ bond). The dihedral angle between the benzene ring and 1,4–diazabutadiene plane is 78.3 (2)°. Niether hydrogen bonding nor aromatic stacking are observed in the crystal structure.

### 2. Experimental

Formic acid (0.5 ml) was added to a stirred solution of 2,3–butanedione (0.09 g, 1.00 mmol) and 4,6–dibromo–2,3–dimethylaniline (0.61 g, 2.2 mmol) in ethanol (10 ml) (Fig. 2). The mixture was refluxed for 24 h, and then cooled and the precipitate was separated by filtration. The solid was recrystallized from *Et*OH/CH_2_Cl_2_ (*v*:*v* = 10:1), washed with cold ethanol and dried under vacuum to give the title compound1. Yield is 0.50 g (82%). Crystals suitable for X–ray structure determination were grown from a cyclohexane/dichloromethane (*v*:*v* = 1:2) solution. Anal. Calcd. for C_20_H_20_Br_4_N_2_: C, 39.51; H, 3.32; Br, 52.57; N, 4.61. Found: C, 39.49; H, 3.31; Br, 52.60 N, 4.60.

### 3. Refinement

All hydrogen atoms were placed in calculated positions with C—H distances of 0.93Å and 0.96Å for aryl and methyl H atoms. They were included in the refinement in a riding model approximation, respectively. The H atoms were assigned *U*_iso_ = 1.2*U*_eq_(C) for aryl H and *U*_iso_ = 1.5*U*_eq_(C) for methyl H.

### Crystal data

**Table:**

C_20_H_20_Br_4_N_2_	*F*(000) = 588
*M**_r_* = 607.98	*D*_x_ = 1.922 Mg m^−^^3^
Monoclinic, *P*2_1_/*n*	Cu *K*α radiation, λ = 1.5418 Å
*a* = 5.5582 (6) Å	Cell parameters from 2210 reflections
*b* = 12.8881 (15) Å	θ = 3.4–70.4°
*c* = 14.8377 (11) Å	µ = 9.40 mm^−^^1^
β = 98.782 (8)°	*T* = 150 K
*V* = 1050.43 (19) Å^3^	Block, clear light yellow
*Z* = 2	0.29 × 0.17 × 0.16 mm

### Data collection

**Table:**

Agilent SuperNova (Dual, Cu at zero, Eos) diffractometer	1921 independent reflections
Radiation source: SuperNova (Cu) X-ray Source	1777 reflections with *I* > 2σ(*I*)
Mirror monochromator	*R*_int_ = 0.029
Detector resolution: 16.0733 pixels mm^-1^	θ_max_ = 68.2°, θ_min_ = 4.6°
ω–scans	*h* = −6→4
Absorption correction: multi-scan (*CrysAlis PRO*; Agilent, 2013)	*k* = −15→15
*T*_min_ = 0.688, *T*_max_ = 1.000	*l* = −17→17
4675 measured reflections	

### Refinement

**Table:**

Refinement on *F*^2^	Primary atom site location: structure-invariant direct methods
Least-squares matrix: full	Secondary atom site location: difference Fourier map
*R*[*F*^2^ > 2σ(*F*^2^)] = 0.044	Hydrogen site location: inferred from neighbouring sites
*wR*(*F*^2^) = 0.135	H-atom parameters constrained
*S* = 1.11	*w* = 1/[σ^2^(*F*_o_^2^) + (0.0802*P*)^2^ + 2.2115*P*] where *P* = (*F*_o_^2^ + 2*F*_c_^2^)/3
1921 reflections	(Δ/σ)_max_ < 0.001
121 parameters	Δρ_max_ = 0.70 e Å^−^^3^
0 restraints	Δρ_min_ = −1.04 e Å^−^^3^

### Special details

**Table:**

Geometry. All s.u.'s (except the s.u. in the dihedral angle between two l.s. planes) are estimated using the full covariance matrix. The cell s.u.'s are taken into account individually in the estimation of s.u.'s in distances, angles and torsion angles; correlations between s.u.'s in cell parameters are only used when they are defined by crystal symmetry. An approximate (isotropic) treatment of cell s.u.'s is used for estimating s.u.'s involving l.s. planes.
Refinement. Refinement of *F*^2^ against ALL reflections. The weighted *R*–factor *wR* and goodness of fit *S* are based on *F*^2^, conventional *R*–factors *R* are based on *F*, with *F* set to zero for negative *F*^2^. The threshold expression of *F*^2^ > σ(*F*^2^) is used only for calculating *R*–factors(gt) *etc*. and is not relevant to the choice of reflections for refinement. *R*–factors based on *F*^2^ are statistically about twice as large as those based on *F*, and *R*–factors based on ALL data will be even larger.

### Fractional atomic coordinates and isotropic or equivalent isotropic displacement parameters (Å^2^)

**Table:**

	*x*	*y*	*z*	*U*_iso_*/*U*_eq_	
Br2	0.84461 (11)	0.31214 (5)	0.68427 (4)	0.0431 (2)	
N1	0.5517 (8)	0.5134 (4)	0.6182 (3)	0.0327 (9)	
C2	0.2754 (9)	0.4881 (4)	0.8311 (3)	0.0269 (9)	
C3	0.6169 (9)	0.3862 (4)	0.7425 (3)	0.0277 (10)	
C4	0.4939 (9)	0.4723 (4)	0.7012 (3)	0.0275 (10)	
C5	0.3261 (10)	0.5238 (4)	0.7464 (3)	0.0296 (10)	
C6	0.4633 (10)	0.4739 (4)	0.5417 (3)	0.0341 (11)	
C7	0.5762 (9)	0.3504 (4)	0.8271 (3)	0.0293 (10)	
H7	0.6616	0.2939	0.8548	0.035*	
C1	0.1862 (10)	0.6189 (4)	0.7022 (3)	0.0341 (11)	
H1A	0.0200	0.5999	0.6813	0.051*	
H1B	0.2604	0.6422	0.6515	0.051*	
H1C	0.1912	0.6736	0.7463	0.051*	
C8	0.2980 (12)	0.3814 (5)	0.5268 (3)	0.0438 (14)	
H8A	0.1479	0.4010	0.4898	0.066*	
H8B	0.2654	0.3560	0.5845	0.066*	
H8C	0.3751	0.3278	0.4964	0.066*	
C9	0.0867 (10)	0.5414 (4)	0.8773 (4)	0.0378 (12)	
H9A	0.1616	0.5968	0.9148	0.057*	
H9B	0.0170	0.4924	0.9146	0.057*	
H9C	−0.0388	0.5691	0.8321	0.057*	
C10	0.4039 (9)	0.4017 (4)	0.8689 (3)	0.0277 (10)	
Br1	0.34078 (11)	0.34478 (4)	0.98167 (3)	0.0379 (2)	

### Atomic displacement parameters (Å^2^)

**Table:**

	*U*^11^	*U*^22^	*U*^33^	*U*^12^	*U*^13^	*U*^23^
Br2	0.0473 (4)	0.0546 (4)	0.0317 (3)	−0.0012 (3)	0.0199 (3)	−0.0110 (2)
N1	0.043 (2)	0.043 (2)	0.0129 (18)	−0.0155 (19)	0.0064 (16)	0.0029 (17)
C2	0.035 (2)	0.030 (2)	0.017 (2)	−0.0044 (19)	0.0075 (17)	−0.0020 (17)
C3	0.038 (2)	0.032 (2)	0.015 (2)	−0.005 (2)	0.0109 (17)	−0.0075 (18)
C4	0.042 (3)	0.032 (2)	0.0096 (19)	−0.012 (2)	0.0066 (17)	−0.0023 (17)
C5	0.047 (3)	0.026 (2)	0.015 (2)	−0.006 (2)	0.0046 (19)	0.0011 (18)
C6	0.047 (3)	0.044 (3)	0.013 (2)	−0.016 (2)	0.0102 (19)	0.001 (2)
C7	0.042 (3)	0.025 (2)	0.022 (2)	−0.003 (2)	0.007 (2)	−0.0012 (17)
C1	0.053 (3)	0.029 (2)	0.019 (2)	−0.004 (2)	0.001 (2)	0.0107 (19)
C8	0.061 (4)	0.058 (3)	0.013 (2)	−0.031 (3)	0.007 (2)	0.000 (2)
C9	0.045 (3)	0.042 (3)	0.029 (3)	0.005 (2)	0.015 (2)	−0.003 (2)
C10	0.044 (3)	0.030 (2)	0.0102 (18)	−0.004 (2)	0.0077 (17)	0.0000 (17)
Br1	0.0605 (4)	0.0407 (4)	0.0159 (3)	0.0013 (2)	0.0171 (2)	0.00654 (19)

### Geometric parameters (Å, º)

**Table:**

Br2—C3	1.895 (5)	C7—H7	0.9300
N1—C4	1.422 (6)	C7—C10	1.385 (7)
N1—C6	1.273 (6)	C1—H1A	0.9600
C2—C5	1.406 (6)	C1—H1B	0.9600
C2—C9	1.504 (7)	C1—H1C	0.9600
C2—C10	1.395 (7)	C8—H8A	0.9600
C3—C4	1.395 (7)	C8—H8B	0.9600
C3—C7	1.388 (6)	C8—H8C	0.9600
C4—C5	1.397 (7)	C9—H9A	0.9600
C5—C1	1.543 (7)	C9—H9B	0.9600
C6—C6^i^	1.518 (9)	C9—H9C	0.9600
C6—C8	1.501 (7)	C10—Br1	1.908 (4)
			
C6—N1—C4	121.1 (4)	C5—C1—H1B	109.5
C5—C2—C9	120.5 (5)	C5—C1—H1C	109.5
C10—C2—C5	117.4 (4)	H1A—C1—H1B	109.5
C10—C2—C9	122.1 (4)	H1A—C1—H1C	109.5
C4—C3—Br2	121.1 (3)	H1B—C1—H1C	109.5
C7—C3—Br2	117.2 (4)	C6—C8—H8A	109.5
C7—C3—C4	121.6 (4)	C6—C8—H8B	109.5
C3—C4—N1	121.1 (4)	C6—C8—H8C	109.5
C3—C4—C5	119.1 (4)	H8A—C8—H8B	109.5
C5—C4—N1	119.6 (4)	H8A—C8—H8C	109.5
C2—C5—C1	118.9 (5)	H8B—C8—H8C	109.5
C4—C5—C2	120.9 (4)	C2—C9—H9A	109.5
C4—C5—C1	120.2 (4)	C2—C9—H9B	109.5
N1—C6—C6^i^	115.8 (6)	C2—C9—H9C	109.5
N1—C6—C8	126.4 (4)	H9A—C9—H9B	109.5
C8—C6—C6^i^	117.8 (5)	H9A—C9—H9C	109.5
C3—C7—H7	121.1	H9B—C9—H9C	109.5
C10—C7—C3	117.8 (5)	C2—C10—Br1	120.6 (4)
C10—C7—H7	121.1	C7—C10—C2	123.2 (4)
C5—C1—H1A	109.5	C7—C10—Br1	116.2 (4)
			
Br2—C3—C4—N1	−6.1 (6)	C5—C2—C10—C7	−0.1 (7)
Br2—C3—C4—C5	179.4 (3)	C5—C2—C10—Br1	−178.0 (4)
Br2—C3—C7—C10	−177.8 (4)	C6—N1—C4—C3	82.4 (7)
N1—C4—C5—C2	−176.5 (4)	C6—N1—C4—C5	−103.1 (6)
N1—C4—C5—C1	5.7 (7)	C7—C3—C4—N1	174.7 (4)
C3—C4—C5—C2	−1.9 (7)	C7—C3—C4—C5	0.2 (7)
C3—C4—C5—C1	−179.7 (4)	C9—C2—C5—C4	−177.4 (5)
C3—C7—C10—C2	−1.5 (7)	C9—C2—C5—C1	0.4 (7)
C3—C7—C10—Br1	176.5 (4)	C9—C2—C10—C7	179.1 (5)
C4—N1—C6—C6^i^	179.2 (6)	C9—C2—C10—Br1	1.2 (7)
C4—N1—C6—C8	−1.5 (10)	C10—C2—C5—C4	1.8 (7)
C4—C3—C7—C10	1.5 (7)	C10—C2—C5—C1	179.6 (4)

Symmetry code: (i) −*x*+1, −*y*+1, −*z*+1.
